# Data-Driven MOX Chemosensing for Beer Discrimination: Towards Rapid Food Quality Screening

**DOI:** 10.3390/mi17070840

**Published:** 2026-07-15

**Authors:** Luca Manini, Elisabetta Poeta, Estefanía Núñez-Carmona, Veronica Sberveglieri

**Affiliations:** 1 Nano Sensor Systems S.r.l. (NASYS), Via Alfonso Catalani, 9, 42121 Reggio Emilia, Italy; veronica.sberveglieri@cnr.it; 2 Department of Mathematical, Physical and Computer Sciences, University of Parma, Parco Area Delle Scienze 7/A, 43124 Parma, Italy; 3 Institute of Bioscience and Bioresources (CNR-IBBR), National Research Council, URT-Re, Via J.F. Kennedy, 17/i, 42124 Reggio Emilia, Italy; elisabettapoeta@cnr.it

**Keywords:** semiconducting metal oxide sensors, volatile organic compounds, non-invasive technology, machine learning, lager beer

## Abstract

Beer quality assessment increasingly requires rapid and scalable analytical tools for product discrimination and authenticity control. In this study, a data-driven metal oxide semiconductor (MOX) chemosensing approach was investigated for the discrimination of commercial lager beers with different alcohol contents and brands. Alcoholic and alcohol-free beer samples from four commercial brands were analyzed using a six-element SnO_2_-based MOX sensor array, and the resulting response patterns were classified using supervised machine-learning algorithms. Headspace solid-phase microextraction gas chromatography–mass spectrometry (HS-SPME-GC–MS) was employed as a reference technique to characterize volatile organic compound profiles and support the interpretation of sensor-based fingerprints. GC–MS analysis highlighted a shared volatile backbone dominated by fermentation-related compounds, while also revealing brand- and category-dependent differences in VOC distribution. The MOX sensor array captured these differences as multidimensional volatile fingerprints. Machine-learning models achieved high classification performance in brand-matched alcoholic versus alcohol-free comparisons, with balanced accuracy ranging from 0.937 to 1.000, while brand discrimination within the same category reached balanced accuracy values of 0.875 (alcoholic) and 0.933 (alcohol-free). These results highlight MOX-based chemosensing combined with data-driven analysis as a rapid, portable platform for beer discrimination, with applications in food quality screening, authenticity assessment, and at-line monitoring.

## 1. Introduction

In recent years, the brewing sector has undergone a profound transformation, characterized not only by an increasing diversification of products but also by a redefinition of quality paradigms driven by emerging consumption patterns. Within this framework, low- and non-alcoholic beers represent one of the fastest-growing segments, supported by rising health awareness, changing lifestyles, and increasingly stringent regulatory constraints [1]. However, alongside this transition, maintaining high sensory quality—particularly in terms of aromatic complexity and product identity—has emerged as a critical challenge for the industry [2].

The aroma profile of beer results from a complex network of volatile organic compounds (VOCs), generated and modulated throughout the different stages of the production process [3]. Dealcoholization technologies, while essential for the development of alcohol-free products, introduce significant perturbations to the volatilome, altering delicate chemical equilibria and often compromising organoleptic properties [4]. The ability to monitor and interpret these variations in a rapid, accurate, and non-destructive manner therefore represents a key technological challenge.

Advanced chromatographic techniques, such as gas chromatography coupled with mass spectrometry (GC–MS), are considered the gold standard for VOC analysis due to their high sensitivity and molecular identification capabilities [5]. However, their applicability in dynamic industrial environments is limited by high instrumentation costs, lengthy analysis times, and the requirement for highly skilled personnel, particularly in contexts where rapid and in-line solutions are needed.

In this scenario, gas sensing technologies based on metal oxide (MOX) sensors are emerging as promising alternative tools with applications spanning diverse domains, from food quality monitoring to medical diagnostics and healthcare gas sensing [6,7,8]. Unlike traditional analytical approaches, these systems do not aim at the selective identification of individual compounds but rather at capturing complex patterns of the volatilome, translating them into multidimensional olfactory fingerprints. This paradigm, based on the global analysis of sensor responses, has proven particularly effective in classification, authentication, and quality control applications, including raw material and product quality assessment across the food and beverage sector [9]. Within the beer sector specifically, recent studies have applied electronic nose and sensor-based systems to product quality assessment, such as shelf-life prediction of beer through volatile profile monitoring [10], as well as deep learning-based approaches, including convolutional neural networks, for beer identification via portable electronic nose systems [11].

Despite the growing interest in sensor-based technologies within the agri-food sector, a fundamental issue remains unresolved: the correlation between sensor responses and the actual chemical composition of the sample. In this context, the integration of GC–MS and MOX-based systems represents a strategic approach, combining analytical accuracy with operational speed, providing a more comprehensive interpretation of the volatilome, and facilitating the translation of chemical data into actionable real-time information.

In the brewing field, such integration is particularly relevant for discriminating beers with different alcohol contents, as well as different brands and production types. This capability is not only essential for quality control but is also increasingly important for traceability, authenticity assessment, and fraud prevention in a highly competitive global market [12].

At the same time, the use of rapid sensing systems opens new perspectives across the entire production chain, from fermentation monitoring to shelf-life evaluation. In this context, MOX sensors stand out due to their low cost, portability, and ease of integration into embedded platforms, making them well-suited for in-line and distributed monitoring applications.

In light of these considerations, the present study aims to investigate an integrated GC–MS and MOX-based sensing approach for the discrimination of commercial beers differing in alcohol content (alcoholic vs. alcohol-free) and brand. HS-SPME-GC–MS was first employed to characterize the volatile organic compound profiles of the selected samples, providing a chemical reference against which the discriminative capability of a six-element MOX sensor array was then evaluated through a dedicated feature-based machine-learning framework. Particular attention was further devoted to assessing the feasibility of rapid, short-exposure discrimination, as a step toward embedded, in-line quality-screening applications in the brewing sector. All investigated samples belonged to the same beer category, i.e., lager, produced using comparable raw materials and fermentation processes and thus sharing broadly similar volatile organic compound profiles [13,14]. This intrinsic similarity increases the complexity of the classification task, as it reduces pronounced compositional differences and challenges the discriminative capability of the sensing system, thereby allowing the assessment to focus on subtle variations in aroma profiles rather than on macroscopic differences between distinct beer types [15].

## 2. Materials and Methods

### 2.1. Sample Preparation

Beer samples were selected from four widely recognized commercial brands available on the Italian market. Samples were purchased weekly from a local store over the entire five-month sampling period and analyzed within the following days of each corresponding week. Samples were stored at room temperature between purchase and analysis without any further treatment, consistent with routine product acquisition practices, thereby capturing the natural batch turnover of commercially available stock rather than relying on a single production lot. For each brand, one alcoholic beer and its corresponding alcohol-free commercial variant were analyzed: Heineken Original (HNK) and Heineken 0.0 (HNK_0), Peroni Nastro Azzurro Originale (NA) and Peroni Nastro Azzurro 0.0% (NA_0), Birra Moretti Ricetta Originale (MRT) and Birra Moretti La Zero (MRT_0), Forst Premium (FRST) and Forst 0.0% (FRST_0). The sample codes reported in parentheses were adopted consistently throughout the study. All samples were selected within the same beer category, i.e., lager [16].

### 2.2. Determination of Volatile Compound by GC-MS

To provide chemical context for the interpretation of the MOX sensor array responses, the volatile profiles of the beer samples were independently characterized using headspace solid-phase microextraction (HS-SPME) coupled with gas chromatography–mass spectrometry (GC–MS).

For each analysis, 5 mL of sample were transferred into a sterile glass vial and equilibrated at 50 °C for 16 min using an ICF 120 incubator (ARGO LAB, Giorgio Bormac S.r.l., Carpi, Italy). Headspace extraction was then performed using a divinylbenzene/carboxen/polydimethylsiloxane (DVB/CAR/PDMS, 50/30 μm) fiber (Supelco, Bellefonte, PA, USA), the extraction step was conducted at 50 °C for 30 min.

Following extraction, the analytes were analyzed using a GC-2020 gas chromatograph coupled with an MS-QP2020 mass spectrometer (Shimadzu, Kyoto, Japan). The fiber was placed in the GC injector port for 6 min at 240 °C in direct mode leading to the thermal desorption of the volatile compounds. Gas chromatographic separation was achieved using a low-polarity stationary phase MEGA-5MS column (25 m × 0.25 mm internal diameter × 0.25 μm film thickness) from Agilent Technologies (Santa Clara, CA, USA). Hydrogen gas with 99.99% purity, supplied by the GENius PF500 system (FullTech Instruments Srl, Rome, Italy), was employed as the carrier gas, at 35.7 kPa, 2.2 mL/min flow, 87.4 cm/s linear velocity, and 4.0 mL/min purge flow. The column oven temperature program consisted of an initial temperature of 40 °C for 3 min, a gradient of 5 °C/min to 150 °C, followed by a gradient of 15 °C/min to 200 °C, and a final hold at 200 °C for 2 min. The total chromatographic run time was 30 min. Mass spectrometric detection was conducted under electron impact (EI) ionization at 70 eV, using the full-scan acquisition mode within the 40–350 *m*/*z* range. The transfer line and ion source were both held at a constant temperature of 200 °C. Data acquisition was performed in the total ion current (TIC) mode at interval of 0.3 s. The detector temperature was set at 240 °C. Compound identification was performed by comparison of acquired mass spectra with three reference libraries (Nist11, Nist 11b, and FFNSC2) with automatic peak integration was carried out using peak area as the quantification parameter, a minimum of 70 peaks with area values ≥ 500 AMU were considered for analysis. Integration parameters included a slope of 100/min, peak width of 2 s, drift of 0/min, and doubling time (T.DBL) of 1000 min. No signal smoothing was applied. Volatile compounds were quantified in terms of relative abundance, expressed as a percentage of the total GC peak area [17].

### 2.3. MOX Sensor Array Platform

All measurements were performed using an S3+ device (Nano Sensor Systems Srl, Reggio Emilia, Italy), equipped with an array of six metal oxide semiconductor (MOX) gas sensors. The system consists of a sample container, a sensor chamber, a diaphragm pump, three solenoid valves, and a carbon filter. The sensing elements were based on tin dioxide (SnO_2_) as the active semiconducting material, used either in its pristine form or modified with selected noble-metal additives to diversify the surface reactivity of the array [18]. Specifically, the sensor array included SnO_2_-based elements with different surface functionalization involving palladium (Pd), platinum (Pt), and gold (Au) as shown in Table 1. This controlled variation in surface composition is designed to expand the chemical response space of the array, thereby improving its capability to generate distinctive and informative responses in the presence of complex VOC mixtures [19]. The sensors were operated at a constant working temperature of 500 °C, maintained by integrated platinum-based micro-heaters.

The sensor chamber (11 × 6.5 × 1.3 cm) was designed to promote uniform airflow over the sensing surfaces while limiting the influence of external environmental fluctuations. The MOX sensors were linearly arranged within the chamber to ensure comparable exposure to the sample headspace, thereby providing controlled measurement conditions and improving the reproducibility of sensor–analyte interactions [20].

Gas handling is achieved through a dynamic fluidic circuit composed of a diaphragm pump (model NMP05B, KNF, Milan, Italy), polyurethane tubing, a solenoid valve (model K000-303-K11M, Camozzi Group S.p.A., Brescia, Italy), and an activated carbon filter placed along the inlet line to provide purified reference air and reduce the contribution of background contaminants. The solenoid valve, installed upstream of the sensing chamber, regulates the airflow delivered by the pump, with a maximum flow rate of 250 sccm, thus enabling controlled and repeatable exposure conditions. The valve was responsible for switching the inlet line between two sources: the activated carbon filter, delivering purified reference air to the sensing chamber, and the sample container, directing the beer headspace into the sensing chamber for analysis. To account for environmental variability, the system is equipped with a temperature and humidity sensor (AM2320, Guangzhou Aosong Electronics Co., Ltd., Guangzhou, China), which continuously monitors ambient temperature (T, °C) and relative humidity (RH, %). These parameters are used to ensure that all measurements are performed under comparable environmental conditions, thereby minimizing potential biases in the sensor responses.

### 2.4. Operational Configuration and Data Acquisition

For each measurement, 100 mL of beer were transferred into a 250 mL glass vessel, which was closed during the conditioning step to allow headspace accumulation. Before analysis, the samples were conditioned at 50 °C for 16 min to standardize headspace generation and improve the reproducibility of sensor exposure. The conditioning time was selected based on preliminary tests, as it provided stable and repeatable sensor responses while maintaining the protocol compatible with a controlled laboratory screening workflow. After conditioning, the accumulated headspace was dynamically sampled by the MOX device through its fluidic circuit and delivered to the sensor chamber by the integrated pump.

**Figure 1:**
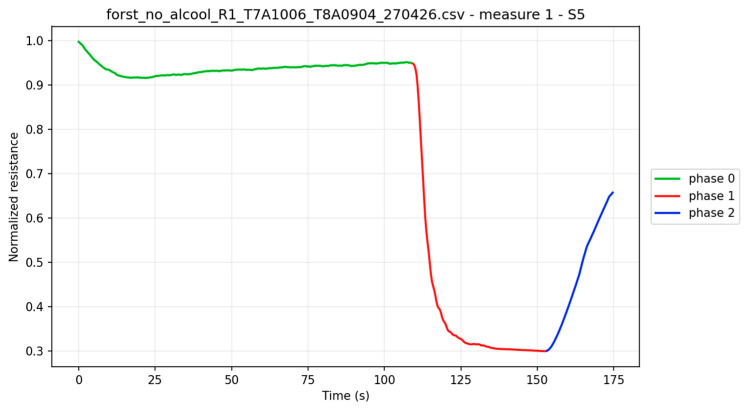
Representative normalized resistance response of sensor S5 during one measurement cycle performed on sample FRST_0. The cycle comprises three sequential phases: Phase A (green), Phase B (red) and Phase C (blue).

Each measurement cycle consisted of three sequential phases. In the initial phase (Phase A), filtered air was continuously flushed over the sensor array to establish and record a stable baseline. This was followed by the sampling phase (Phase B), during which the sample headspace was introduced into the sensing chamber for analysis. In the final phase (Phase C), the sensors were again exposed to filtered air to promote signal recovery. Raw response profiles of all six sensors across representative samples of each brand are reported in Figures S1 and S2, for alcohol-free and alcoholic beers, respectively. As a representative example, Figure 1 shows the normalized resistance response of a single sensor (S5) during one measurement cycle.

To improve dataset robustness and account for experimental variability, beer samples were analyzed over multiple days, with multiple measurement cycles acquired for each sample, each comprising the full sequence of Phases A, B, and C.

Different acquisition times were used according to the beer category to account for the different response dynamics observed during preliminary measurements. Alcoholic beers were analyzed using 110 s of baseline stabilization, 10 s of headspace exposure, and 21 s of recovery; whereas, alcohol-free beers were measured using 110 s, 43 s, and 21 s for the same phases, respectively. Each measure lasted 2 min and 21 s for the alcoholic beer samples and 2 min and 54 s for the alcohol-free sample. This choice reflected the faster headspace-induced response observed for alcoholic beers, likely related to the higher contribution of ethanol and other volatile constituents [21].

For each classification scenario, all samples were compared using sensor signals extracted over an equivalent sampling duration. In brand-matched alcoholic versus alcohol-free comparisons, only the portion of the alcohol-free signal corresponding to the sampling window used for the respective alcoholic beer was retained, ensuring that both classes were evaluated over the same temporal response interval. This choice reflects the distinct headspace-induced response dynamics observed for alcoholic beers, and was also exploited to assess the feasibility of rapid discrimination, by evaluating whether the early sensor response contained sufficient information for sample classification. Restricting the alcohol-free signal to this shorter window may exclude potentially discriminative information present in its later response phase; however, this information was not excluded from the study overall, as the multiclass brand-discrimination analysis for alcohol-free beers was performed using the full native sampling window, allowing later-phase response dynamics to be exploited in that context.

### 2.5. Data Processing

Raw sensor data were organized as individual CSV files, each corresponding to a single experimental acquisition. Each file contained multiple measurement cycles, recorded as time-series signals from the six MOX sensors and structured according to the experimental sequence described above. For machine-learning analysis, the sampling phase was selected as the most informative region, as it corresponds to the direct exposure of the sensor array to the sample headspace [22]. This phase was therefore used to extract the response patterns associated with the volatile fraction of each sample. Prior to feature extraction, sensor responses were preprocessed to reduce acquisition-dependent variability and improve comparability across measurements, specifically, each sensor signal was normalized with respect to its corresponding baseline within the same measurement cycle. This transformation converted raw resistance values into relative response profiles, reducing the influence of baseline offsets and intrinsic differences among sensing elements [23]. After preprocessing, each normalized sensor response was converted into a set of descriptive features extracted from the sampling phase. The feature set included internally defined statistical, temporal, derivative-based, integral, variability, entropy, energy-related, and signal-shape descriptors, hereafter referred to as standard features. These custom descriptors were complemented by additional features generated using established time-series feature extraction libraries, including catch22, tsfresh, and tsfel [24,25,26]. This combined feature space was designed to retain both interpretable response characteristics and more complex temporal patterns encoded in the sensor signals. Such an approach is important because classification tasks may involve samples sharing a largely similar volatile backbone, in which case classification may depend on subtle differences in sensor response dynamics [27].

Feature selection was then applied to reduce the dimensionality of the high-dimensional candidate feature space generated from the MOX time-series responses. The procedure combined low-variance filtering with mutual-information-based ranking of feature relevance with respect to the class labels. Multiple combinations of variance and percentile thresholds were evaluated, ranging from 0.1 to 0.9 with increments of 0.1. For each combination, the most informative descriptors were retained while enforcing a minimum number of selected features. Among the candidate feature subsets, the pipeline prioritized those showing better preliminary classification performance, while also accounting for the presence of highly correlated features, before proceeding to full classifier training.

### 2.6. Supervised Model Development and Evaluation

Before model training, the dataset was partitioned at the acquisition-file level into a training set (70%) and a validation set (30%) using stratified sampling to preserve the class distribution of each classification task. Feature selection was performed using only the training set. The same training set was then further divided into an internal training subset (70%) and an internal test subset (30%) using a group-based split. The internal training subset was used for classifier fitting and hyperparameter optimization; whereas, the internal test subset was used for model assessment during development. The validation set was kept separate from feature selection, hyperparameter optimization, and model fitting, and was used after model development to evaluate candidate models and generate the final model ranking [28].

A broad panel of supervised classification algorithms was evaluated to compare different modeling strategies. The candidate model pool included logistic regression (LgRg), support vector machines (SVC), polynomial-kernel support vector machines (PSVM), radial-basis-function support vector machines (RSVM), k-nearest neighbors (KNN), decision trees (DT), random forests (RF), Extra Trees (ExTr), gradient boosting (GB), AdaBoost (Ada), XGBoost (XGB), naive Bayes (NB), quadratic discriminant analysis (QDA), multilayer perceptron classifiers (MLP), a KMeans-based logistic regression pipeline (KMLR), a linear-discriminant-analysis/SVC pipeline (LSVC), and voting-based ensemble models. For each feature subset generated during the feature-selection stage, the classifiers were trained and evaluated using the same data partitioning strategy, enabling systematic comparison across feature configurations and algorithm families. Feature standardization was included within the machine-learning pipeline as a preprocessing step before classifier training. The scaler was fitted only on the internal training subset and subsequently applied to the internal test subset and to the validation set, preventing statistical information from the evaluation data from being transferred into the model-fitting process. Model performance was assessed separately on the internal test subset and on the validation set. The final model ranking was primarily based on balanced accuracy on the validation set, with macro-F1 and internal test balanced accuracy used as secondary criteria. Balanced accuracy was selected as the main ranking metric because it accounts for class-wise performance and is more informative than overall accuracy when the number of observations per class is not perfectly balanced [29,30]. Additional metrics, including accuracy, macro-precision, macro-recall, macro-F1, and ROC AUC when available, were used to provide a more comprehensive evaluation of classifier performance.

### 2.7. Experimental Classification Scenarios

The workflow was applied to complementary classification tasks addressing both alcohol-status recognition and brand-level discrimination. Binary classification was first performed to distinguish alcoholic and alcohol-free variants within the same brand, with each brand-matched comparison treated as an independent case study. Specifically, four binary comparisons were considered: HNK vs. HNK_0; NA vs. NA_0; MRT vs. MRT_0 and FRST vs. FRST_0. In addition, two multiclass classification tasks were performed separately on alcoholic beers (HNK, NA, MRT, and FRST) and alcohol-free beers (HNK_0, NA_0, MRT_0, and FRST_0), in order to assess whether MOX sensor response patterns could discriminate among brands within the same alcohol category. For each task, data partitioning, feature selection, model development, validation, and final ranking were carried out independently, ensuring that each classification scenario was evaluated under task-specific conditions.

## 3. Results and Discussion

### 3.1. GC-MS Analysis

GC–MS analysis was performed to provide chemical support for the interpretation of the volatile fingerprints recorded by the MOX sensor array. All beer samples, including both alcoholic and alcohol-free variants, were characterized by HS-SPME-GC–MS.

**Figure 2:**
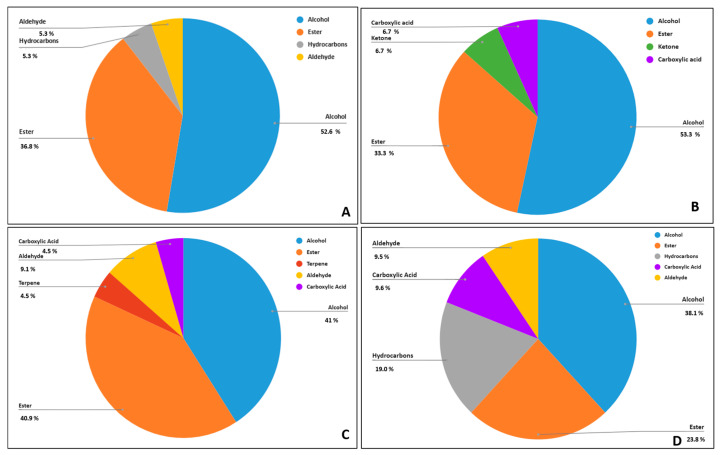
Distribution of chemical classes of volatile compounds identified by GC–MS in alcoholic beer samples. Pie charts show the relative contribution of each chemical class in the four commercial lager beers analyzed: (**A**) Nastro Azzurro, (**B**) Forst, (**C**) Heineken, and (**D**) Moretti.

**Figure 3:**
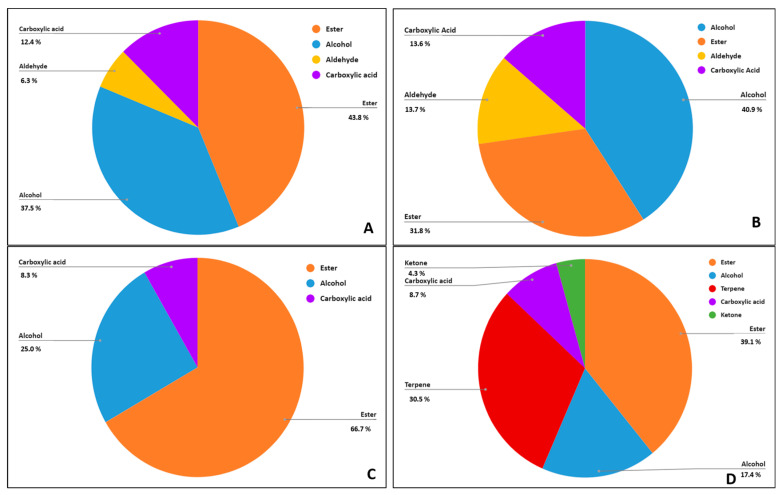
Chemical class composition of volatile compounds detected by GC–MS in non-alcoholic beer samples. Each pie chart illustrates the proportion of the main chemical families identified in the selected commercial lager beers: (**A**) Nastro Azzurro 0, (**B**) Forst 0, (**C**) Heineken 0, and (**D**) Moretti 0.

The resulting profiles were interpreted as relative volatile fingerprints rather than absolute quantitative measurements; accordingly, they were used to describe differences in headspace composition and chemical diversity among samples, rather than to determine compound-specific concentrations. Within this framework, the distribution of the detected compounds among chemical classes was used to obtain an overview of the volatilome complexity of the samples (Figure 2 and Figure 3). The retained volatile fraction mainly included compounds commonly associated with beer aroma, such as esters, higher alcohols, organic acids, aldehydes, and minor terpenoid compounds [31].

Fermentation-related VOCs, including ethyl acetate, isoamyl alcohol, isoamyl acetate, ethyl hexanoate, ethyl butyrate, phenylethyl alcohol, and phenylethyl acetate were recurrently detected across the samples (Table S3).

The presence of a shared volatile backbone, mainly represented by fermentation-derived esters and alcohols, is consistent with the fact that all samples belong to the same beer style [32]. However, differences in the relative distribution of both major and minor VOC classes indicated sample-dependent variations in the overall volatile fingerprint.

To further investigate the data from GC-MS, selected representative volatiles were manually assimilated and their relative abundances were used for further multivariate analysis (Table S3). PCA was applied to reduce data dimensionality by projecting the original variables into a smaller set of uncorrelated principal components (PCs) [33]. Separate PCA models were built for alcoholic and alcohol-free beers, rather than directly comparing each alcoholic beer with its alcohol-free counterpart. This choice was made to evaluate brand-dependent differences within each product category while avoiding the confounding effect of the large compositional changes associated with alcohol removal and ethanol content. In this way, the PCA analysis was used to assess whether samples belonging to the same category could still be differentiated on the basis of their volatile fingerprints.

**Figure 4:**
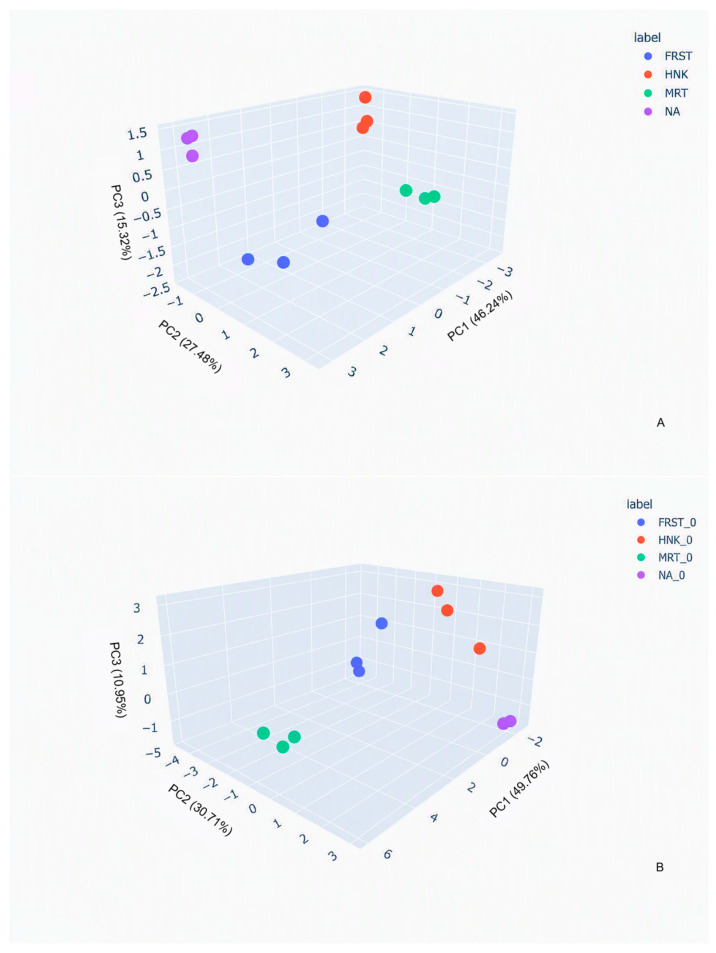
Three-dimensional PCA score plots obtained from GC–MS data for (**A**) alcoholic beer samples and (**B**) alcohol-free beer samples. FRST and FRST_0 are shown in blue, HNK and HNK_0 in red, MRT and MRT_0 in green, and NA and NA_0 in purple.

As shown in Figure 4A, for the alcoholic beer samples, the three-dimensional PCA representation showed that the first three principal components explained 89.04% of the total variance (PC1 = 46.24%, PC2 = 27.48%, PC3 = 15.32%) The score plot showed clear separation among the four brands, suggesting that distinguishable VOC patterns were retained despite the presence of several shared fermentation-related compounds. The loading values of the original variables on the first three principal components are reported in Table S4. Positive and negative loading values indicate opposite directions of association of each compound with the corresponding principal component.

Since sample separation was distributed across the three-dimensional PCA space, the loading analysis was interpreted by considering the compounds with the highest absolute loading values on each principal component. Specifically, the three compounds with the highest absolute loadings for each PC were selected as the main contributors for interpretation. PC1 was mainly associated with variations in ethyl hexanoate, phenethyl acetate, and ethanol. PC2 was primarily influenced by decanoic acid, hexyl acetate, and isoamyl alcohol; whereas, PC3 was mainly associated with isoamyl acetate, ethyl butyrate, and 2-isopropyl-5-methyl-1-heptanol. These compounds should therefore be interpreted as the VOCs most strongly associated with the observed multivariate separation, rather than as exclusive chemical markers of individual beer brands.

Similarly Figure 4B shows the three-dimensional PCA representation obtained from the alcohol-free beer dataset.

The first three principal components explained 91.44% of the total variance (PC1 = 49.76%, PC2 = 30.71%, PC3 = 10.95%). The alcohol-free samples also showed clear clustering according to brand, indicating that brand-level differences in the volatile fingerprint remained detectable despite the reduction in ethanol-related contributions. As for the alcoholic beers, the loading analysis was based on the three compounds with the highest absolute loading values on each principal component. PC1 was mainly associated with variations in limonene, caryophyllene and humulene, PC2 with ethyl acetate, ethyl propionate and isoamyl acetate; and PC3 with phenylethyl acetate, ethyl octanoate and isoamyl alcohol (Table S5). These compounds should likewise be interpreted as the VOCs most strongly associated with the observed multivariate separation, rather than as exclusive markers of individual alcohol-free beer brands. Overall, the GC–MS results indicate that the analyzed beers shared a common volatile profile dominated by fermentation-related compounds, while also displaying brand- and category-dependent differences in the relative distribution of selected VOCs. These differences should not be interpreted as absolute quantitative variations in individual compounds, but rather as changes in the global volatile fingerprint. Such evidence supports the feasibility of using the MOX chemosensor array as a cross-reactive system capable of capturing complex headspace patterns and discriminating beer samples on the basis of their overall VOC composition.

### 3.2. MOX Sensors Array Response

#### 3.2.1. Multivariate Analysis

To further investigate the MOX sensor array response to the volatile fingerprints of the analyzed beer samples, the selected feature subsets were examined according to their sensor origin and complemented with exploratory multivariate analyses. Since feature selection was performed independently for each classification scenario, the number and distribution of selected descriptors varied according to the optimal feature extraction family and the variance and percentile thresholds identified for each specific comparison (Files S6–S11 and Figures S12–S17). Table 2 summarizes, for each analysis, the number of selected descriptors and their distribution across the six sensing elements. Overall, the descriptors were distributed over multiple sensors, indicating that the discriminative information was not confined to a single sensing element but relied on multivariate response patterns generated by the array.

In the brand-matched binary comparisons, the number of selected features ranged from 6 in the NA and NA_0 comparison to 61 features in the FRST and FRST_0 comparison, descriptors were retained from all six sensing elements, although their relative contribution differed among comparisons. A similar behavior was observed in the multiclass tasks, where alcohol brand discrimination involved 77 selected features with a stronger contribution from Tables S4 and S5, File S6; whereas, alcohol-free brand discrimination retained 46 features more evenly distributed across the array. These results suggest that different classification scenarios relied on partially distinct regions of the MOX response space, consistent with the cross-reactive nature of the sensor array and supporting the use of the full MOX array for classification analysis.

Exploratory PCA was then applied to the selected feature space to provide a preliminary visualization of the MOX-derived response patterns (Figures S18–S23). Although class separation was not always sharply defined in the reduced PCA space, partial clustering trends were observed, suggesting that class-related information was retained within the multivariate sensor features. Therefore, PCA was considered only as an exploratory visualization tool; whereas, the actual discriminative performance of the system was assessed through supervised classification models evaluated on the validation set.

#### 3.2.2. Supervised Classification Results

The classification performance of the MOS sensor response patterns was evaluated through two complementary classification scenarios. Binary classification was used to discriminate alcoholic and alcohol-free variants within each brand; whereas, multiclass classification was applied separately to alcoholic and alcohol-free beers to assess brand-level discrimination within each alcohol category. For each scenario, sample distribution across training and validation sets is reported in Table 3 and Table 4.

All brand-matched binary comparisons were performed using the same 10 s sampling-phase window, with the optimal feature-selection and model configuration selected according to balanced accuracy on the validation set.

Table 5 shows the best-performing configurations obtained for the brand-matched binary comparisons. For each comparison, only the top-ranked model is reported, whereas, the complete model rankings and additional performance details are provided in Files S24–S27. Overall, the MOS-derived response patterns enabled reliable discrimination between alcoholic and alcohol-free variants of the same brand, with validation balanced accuracy ranging from 0.937 to 1.000.

Among the binary comparison, the highest validation performance was obtained for MRT vs. MRT_0, where the LgRg + LSVC ensemble, trained on catch22-derived features selected with variance and percentile thresholds of 0.3 and 0.1, achieved a balanced accuracy of 1.00. Comparable performance was observed for FRST vs. FRST_0, where the XGB + LSVC ensemble reached a balanced accuracy of 0.987 using standard features selected with thresholds of 0.7 and 0.1. HNK vs. HNK_0 also showed high discrimination capability, with LSVC trained on standard features selected with thresholds of 0.9 and 0.2 yielding a balanced accuracy of 0.980. A most moderate validation performance was observed for NA vs. NA_0, where the RF + GB ensemble trained on tsfresh features selected with thresholds of 0.9 and 0.8 achieved a balanced accuracy of 0.937.

**Figure 5:**
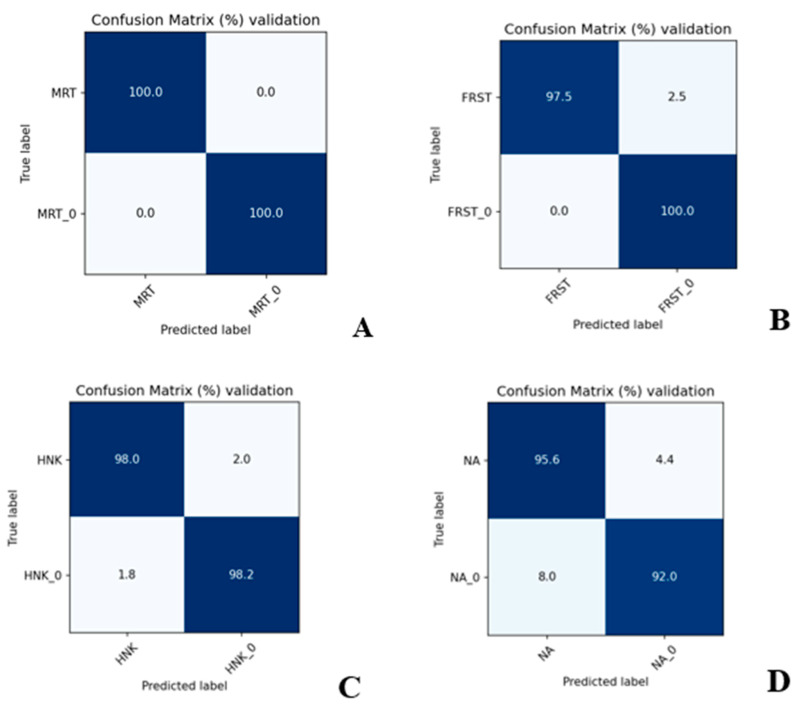
Confusion matrices for the best-performing brand-matched binary classification models: (**A**) Moretti (MRT vs. MRT_0); (**B**) Forst (FRST vs. FRST_0); (**C**) Heineken (HNK vs. HNK_0); (**D**) Nastro Azzurro (NA vs. NA_0). The vertical axis indicates the true beer labels and the horizontal axis indicates the predicted labels.

Figure 5 shows the confusion matrices obtained for the best-performing configuration of each brand-matched binary comparison, providing class-wise information on the distribution of correct predictions and residual misclassifications. The MRT vs. MRT_0 (Figure 5A) comparison showed a fully diagonal pattern, consistent with the balanced accuracy of 1.000, while FRST vs. FRST_0 (Figure 5B) and HNK vs. HNK_0 (Figure 5C) show only limited off-diagonal entries, in agreement with their near-complete validation performance. The NA vs. NA_0 (Figure 5D) comparison exhibits the highest residual misclassification among the binary scenarios, consistent with its more moderate balanced accuracy relative to the other brand-matched comparisons.

The best-performing configurations for the multiclass brand-discrimination scenarios are summarized in Table 6, while the complete model rankings and additional performance details are provided in Files S28 and S29.

The multiclass analyses evaluated whether MOS-derived response patterns could discriminate among the four commercial brands within the same alcohol category. Accordingly, two separate multiclass scenarios were considered: alcoholic beers (HNK, NA, MRT, and FRST) and alcohol-free beers (HNK_0, NA_0, MRT_0, and FRST_0).

**Figure 6:**
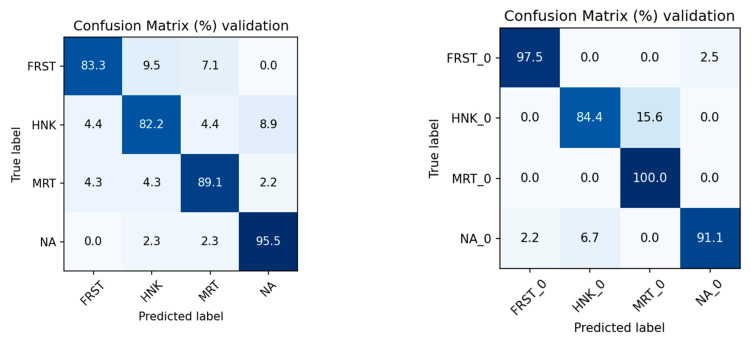
The figure reports the validation confusion matrices obtained for the multiclass classification of alcoholic beers (**left**) and alcohol-free beers (**right**). The vertical axis indicates the true beer labels and the horizontal axis indicates the predicted labels.

For alcoholic beers, the best-performing configuration was based on standard features selected with variance and percentile thresholds of 0.1 and 0.5, respectively. The highest validation performance was achieved by the Extra Trees classifier (ExTr), which reached a validation balanced accuracy of 0.875. For the alcohol-free beer dataset, the optimal configuration also relied on standard features, selected with variance and percentile thresholds of 0.7 and 0.1, respectively. In this case, the best-ranked model was a voting ensemble combining PSVM, LgRg, RF, RSVM, and SVC, which achieved a validation balanced accuracy of 0.933. These results indicate that MOS-derived response patterns were informative for brand-level discrimination within the same alcohol category, with slightly higher validation performance observed for the alcohol-free beer dataset than for the alcoholic beer dataset. This trend is further illustrated by the corresponding confusion matrices Figure 6, which provide class-wise information on correct predictions and residual misclassifications across beer brands.

To further characterize the contribution of individual descriptors to model predictions, permutation importance was computed for the best-performing model of each classification task (Table 5 and Table 6), using balanced accuracy as the scoring metric over 50 permutations per feature. The complete ranking of all retained features per task is reported in Supplementary Tables S30–S35. Overall, the supervised classification results indicate that the MOX sensor array captured both alcohol-status-related and brand-related variations in beer headspace. The higher validation performance observed in the brand-matched binary comparisons suggests that alcoholic and alcohol-free variants of the same brand generated distinguishable sensor fingerprints, likely reflecting differences in the overall volatile response patterns associated with alcohol content. As expected, the multiclass scenarios were more challenging, since the models had to discriminate among four commercial brands within the same alcohol category and therefore characterized by subtler differences in headspace composition. Nevertheless, validation balanced accuracy values obtained for the alcoholic and alcohol-free brand-discrimination analyses indicate that the MOX-derived features retained brand-specific information. These results support the use of the MOX sensor array combined with feature-based machine learning as a rapid fingerprinting approach for beer discrimination at both product-category and brand levels.

## 4. Conclusions

This study demonstrates the potential of MOX sensor technology combined with machine learning as a rapid, non-destructive, and data-driven strategy for discriminating commercial lager beers according to alcohol content and brand. The integration with HS-SPME-GC–MS supported the chemical interpretation of sensor-derived fingerprints, revealing a common volatile backbone dominated by fermentation-related compounds, while also highlighting brand- and category-dependent differences in VOC distribution. The MOX sensor array effectively captured these variations through multidimensional response patterns. Notably, the discrimination between alcoholic and alcohol-free beers of the same brand was achieved using only a 10 s exposure window, confirming the extremely rapid nature of the proposed approach. Despite this short acquisition time, supervised models achieved high classification performance, with balanced accuracy values ranging from 0.937 to 1.000 in binary comparisons. Brand-level classification within the same alcohol category also yielded promising results, indicating that MOX signals retain information related not only to alcohol content but also to product-specific volatile identity. These findings are consistent with the growing body of research investigating sensor-based and machine-learning methodologies for beverage quality assessment and discrimination. The present work contributes to this direction by concurrently addressing alcohol-content and brand-level discrimination within a rapid, short-exposure acquisition framework, corroborated by HS-SPME-GC–MS reference data. Some limitations should nonetheless be acknowledged. The present study was based on a limited number of commercial brands and was carried out within a single-platform, single-laboratory setting. These conditions, while ensuring controlled and consistent acquisition, inevitably constrain the generalizability of the developed models, which may not directly transfer to other product types or measurement systems without further validation. Overall, these findings suggest that the MOX platform could serve as a fast, portable, and scalable screening tool for beer quality assessment, supporting product discrimination, authenticity control, process monitoring, and conformity evaluation. Future work should move toward validating the approach under more realistic industrial conditions, including different production batches, beer styles, storage times, and dealcoholization processes. Further efforts should focus on improving model transferability, reducing the dependence on laboratory-controlled conditions, and developing interpretable correlations between sensor responses, VOC profiles, and sensory attributes. These steps will be essential to support the implementation of MOX-based systems as at-line or in-line tools for real-time quality control in breweries.

## Figures and Tables

**Table 1:** Configuration of the MOX sensing array integrated into the S3+ device. The array is arranged as two three-sensor modules, here indicated as module 1 and module 2.

Sensor Module	Sensing Elements	Sensing Layer Composition
Module 1	S1	SnO_2_ + Pd
Module 1	S2	SnO_2_ + Pt
Module 1	S3	SnO_2_ + Au
Module 2	S4	SnO_2_ + Pd
Module 2	S5	SnO_2_
Module 2	S6	SnO_2_ + Au

**Table 2:** Distribution of selected features across the MOS sensor array for each classification scenario. The table reports the total number of descriptors retained after feature selection and their distribution among the six sensing elements (Figures S1 and S2, Tables S3–S5, File S6). Binary comparisons refer to brand-matched alcoholic versus alcohol-free samples; whereas, multiclass scenarios refer to brand discrimination within alcoholic and alcohol-free beer categories.

Classification Scenario	No. Selected Features	S1	S2	S3	S4	S5	S6
HNK vs. HNK_0	52	11	11	10	7	7	6
NA vs. NA_0	6	1	1	1	1	1	1
MRT vs. MRT_0	44	9	6	7	10	6	6
FRST vs. FRST_0	61	11	11	11	9	9	10
Alcoholic beers	77	15	3	4	19	19	17
Alcohol-free beers	46	7	8	8	7	7	9

**Table 3:** Sample distribution for brand-matched binary classification scenarios. The table reports the number of observations assigned to the training, internal test, and validation sets for each class included in the alcoholic versus alcohol-free comparisons within the same brand.

		Number of Samples			
Comparison	Class	Train	Test	Validation	Total
HNK vs. HNK_0	HNK	75	25	50	150
	HNK_0	85	50	55	190
NA vs. NA_0	NA	55	45	45	145
	NA_0	90	30	50	170
MRT vs. MRT_0	MRT	70	30	55	155
	MRT_0	103	35	63	201
FRST vs. FRST_0	FRST	75	25	40	140
	FRST_0	98	43	70	211

**Table 4:** Sample distribution for multiclass brand-discrimination scenarios. The table reports the number of observations assigned to the training, internal test, and validation sets for each class included in the multiclass analyses, performed separately for alcoholic and alcohol-free beers.

		Number of Samples			
Classification Scenario	Class	Train	Test	Validation	Total
Alcoholic beers	HNK	79	26	45	150
	NA	66	35	44	145
	MRT	84	25	46	155
	FRST	60	38	42	140
Alcohol-free beers	HNK_0	70	35	45	150
	NA_0	65	30	45	140
	MRT_0	75	20	45	140
	FRST_0	65	35	40	140

**Table 5:** Best-performing model configurations for brand-matched binary classification scenarios. For each alcoholic versus alcohol-free comparison within the same brand, the table reports the selected feature extraction family, the variance and percentile thresholds used for feature selection, the top-ranked classifier, and the corresponding balanced accuracy on the validation set.

Classification Scenario	Feature Extraction Family	Feature Selection Thresholds	Best Model	Balanced Accuracy
HNK vs. HNK_0	standard	variance 0.9	LSVC	0.980
		percentile 0.2		
NA vs. NA_0	tsfresh	variance 0.9	RF + GB	0.937
		percentile 0.8		
MRT vs. MRT_0	catch22	variance 0.3	LgRg + LSVC	1.000
		percentile 0.1		
FRST vs. FRST_0	standard	variance 0.7	XGB + LSVC	0.987
		percentile 0.1		

**Table 6:** Best-performing model configurations for multiclass brand-discrimination scenarios. For alcoholic and alcohol-free beer datasets, the table reports the selected feature extraction family, the variance and percentile thresholds used for feature selection, the top-ranked classifier, and the corresponding balanced accuracy on the validation set.

Classification Scenario	Feature Extraction Family	Feature Selection Thresholds	Best Model	Balanced Accuracy
Alcoholic beers	Standard	variance 0.1	ExTr	0.875
		percentile 0.5		
Alcohol-free beers	Standard	variance 0.7	PSVM + LgRg + RF + RSVM + SVC	0.933
		percentile 0.1		

## Data Availability

Data are contained within the article and Supplementary Materials.

## Supplementary Materials

The following supporting information can be downloaded at: https://www.mdpi.com/article/10.3390/mi17070840/s1.
