# Supplementary material for: Data-Driven MOX Chemosensing for Beer Discrimination: Towards Rapid Food Quality Screening
Source: Micromachines (Basel). 2026 Jul 15;17(7):840. doi: 10.3390/mi17070840 (PMC13413555; doi:10.3390/mi17070840)
Supplement: Supplementary file 1 [file micromachines-17-00840-s001.zip › Table S3.pdf]

Table S3: relative contents (%) of volatile components for 8 beers by GC-MS

Volatile compounds	Relative Compounds (mean ± standard deviation)							
	HNK	HNK_0	NA	NA_0	MRT	MRT_0	FRST	FRST_0
Ethanol	45.14±0.68	ND	61.39±1.17	ND	49.83±1.29	ND	61.85±9.37	ND
Isoamyl alcohol	11.63±0.51	21.06±0.57	10.97±0.19	18.38±0.37	10.03±0.84	15.37±0.43	11.33±1.28	16.61±3.61
Ethyl butyrate	ND	1.21±0.64	0.28±0.06	2.08±0.36	0.21±0.03	0.56±0.35	ND	9.16±6.64
Isoamyl acetate	14.4±3.21	48.44±4.76	10.21±0.45	54.76±1.57	11.80±0.37	37.62±0.09	6.68±0.42	36.86±3.98
Ethyl hexanoate	2.16±0.42	2.71±0.31	6.65±0.50	1.72±0.33	5.65±0.51	3.68±0.12	5.07±0.33	2.56±0.75
Phenethyl alcohol	17.20±2.92	5.53±2.04	7.87±0.80	3.91±1.14	18.22±1.19	5.87±0.97	13.62±7.74	21.63±7.10
Citronellyl acetate	ND	ND	0.94±0.09	ND	ND	ND	ND	ND
Phenethyl acetate	5.78±1.03	2.24±0.98	1.27±0.25	ND	2.65±0.40	2.01±0.33	1.42±1.42	0.99±0.11
Hexyl acetate	ND	ND	ND	ND	1.13±0.14	ND	ND	ND
Ethyl Acetate	ND	13.87±0.75	ND	12.39±1.97	ND	10.49±1.64	ND	ND
Ethyl Propionate	ND	1.69±0.40	ND	4.31±0.78	ND	1.36±0.47	ND	ND
Isobutyl acetate	ND	ND	ND	ND	ND	5.58±0.76	ND	ND
Ethyl Octanoate	ND	2.16±1.28	ND	ND	ND	1.99±0.09	ND	2.68±1.32
Dihydrolinalool	ND	ND	ND	ND	ND	4.26±1.24	ND	ND
Citronellol	ND	ND	ND	0.16±0.05	ND	ND	ND	ND
Nerolidol	ND	ND	ND	0.78±0.06	ND	9.96±1.50	ND	ND
Limonene	ND	ND	ND	ND	ND	0.31±0.02	ND	ND
Humulene	ND	ND	ND	ND	ND	0.20±0.02	ND	ND
Caryophyllene	ND	ND	ND	ND	ND	0.09±0.01	ND	ND
Myrcene	ND	ND	ND	ND	ND	0.45±0.11	ND	ND
2,3-Butanediol	ND	1.04±0.45	ND	1.25±0.61	ND	ND	ND	7.76±1.33
Decanoic acid	ND	ND	ND	0.21±0.09	0.44±0.05	ND	ND	1.10±0.61
Methoxy phenyl oxime	ND	ND	ND	ND	ND	ND	ND	0.61±0.05
2,4-diethyl- 1-Heptanol	3.65±0.92	ND	ND	ND	ND	ND	ND	ND
2-Isopropyl-5-methyl-1-heptanol	ND	ND	0.40±0.03	ND	ND	ND	ND	ND
2,6-dimethyl- 2-Octene	ND	ND	ND	ND	ND	0.13±0.03	ND	ND
