# Supplementary material for: Data-Driven MOX Chemosensing for Beer Discrimination: Towards Rapid Food Quality Screening
Source: Micromachines (Basel). 2026 Jul 15;17(7):840. doi: 10.3390/mi17070840 (PMC13413555; doi:10.3390/mi17070840)
Supplement: Supplementary file 1 [file micromachines-17-00840-s001.zip › Table S4.pdf]

Table S4: cumulative loading values of the 12 volatile compounds of alcoholic beer samples for the first three principal components

Volatile compounds	PC1	PC2	PC3
Citronellyl acetate	0.323	-0.167	0.398
Decanoic acid	-0.030	0.541	0.032
Ethanol	0.357	-0.143	-0.289
Ethyl butyrate	0.289	0.220	0.412
Ethyl hexanoate	0.384	0.170	-0.010
Hexyl acetate	-0.028	0.535	0.036
Isoamyl acetate	-0.221	0.068	0.537
Isoamyl alcohol	-0.123	-0.388	-0.047
Phenylethyl alcohol	-0.320	0.230	-0.082
Phenylethyl acetate	-0.382	-0.063	0.273
2,4-diethyl-1-Heptanol	-0.341	-0.231	0.227
2-Isopropyl-5-methyl-1-heptanol	0.323	-0.166	0.401
