# Supplementary material for: Data-Driven MOX Chemosensing for Beer Discrimination: Towards Rapid Food Quality Screening
Source: Micromachines (Basel). 2026 Jul 15;17(7):840. doi: 10.3390/mi17070840 (PMC13413555; doi:10.3390/mi17070840)
Supplement: Supplementary file 1 [file micromachines-17-00840-s001.zip › Table S5.pdf]

Table S5: cumulative loading values of the 21 volatile compounds of alcohol-free beer samples for the first three principal components

Volatile compounds	PC1	PC2	PC3
Caryophyllene	0.295	-0.021	-0.089
Citronellol	-0.109	0.242	-0.357
Decanoic acid	-0.141	-0.287	-0.236
Ethyl acetate	0.068	0.348	0.152
Ethyl butyrate	-0.136	-0.267	-0.186
Ethyl hexanoate	0.241	-0.131	0.169
Ethyl octanoate	0.046	-0.248	0.415
Ethyl propionate	-0.051	0.340	-0.221
Humulene	0.295	-0.020	-0.089
Isoamyl acetate	-0.136	0.334	-0.014
Isoamyl alcohol	-0.141	0.173	0.415
Isobutyl acetate	0.295	-0.020	-0.089
Limonene	0.296	-0.023	-0.085
Myrcene	0.286	-0.017	-0.091
Nerolidol	0.292	-0.004	-0.114
Phenylethyl alcohol	-0.88	-0.332	-0.061
Methoxy phenyl oxime	-0.123	-0.336	-0.089
1,2-Dihydrolinalool	0.290	-0.025	-0.077
2,3-Butanediol	-0.165	-0.307	-0.073
2,6-dimethyl-2-Octene	0.293	-0.022	-0.083
Phenylethyl acetate	0.157	-0.078	0.497
