# Supplementary figures and images for: Data-Driven MOX Chemosensing for Beer Discrimination: Towards Rapid Food Quality Screening
Source: Micromachines (Basel). 2026 Jul 15;17(7):840. doi: 10.3390/mi17070840 (PMC13413555; doi:10.3390/mi17070840)

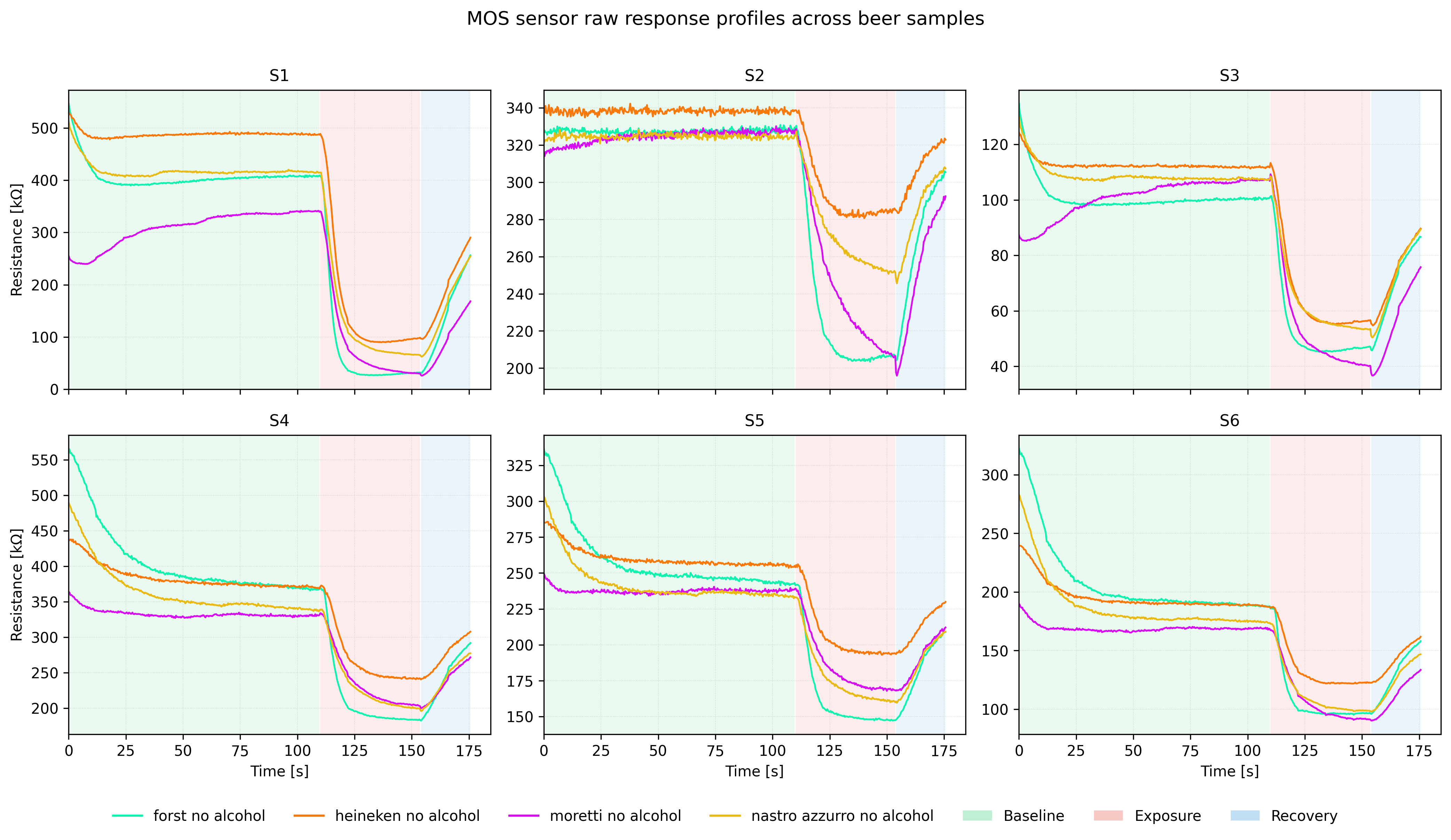

Supplement: Supplementary file 1 [file micromachines-17-00840-s001.zip › Figure S1.png]

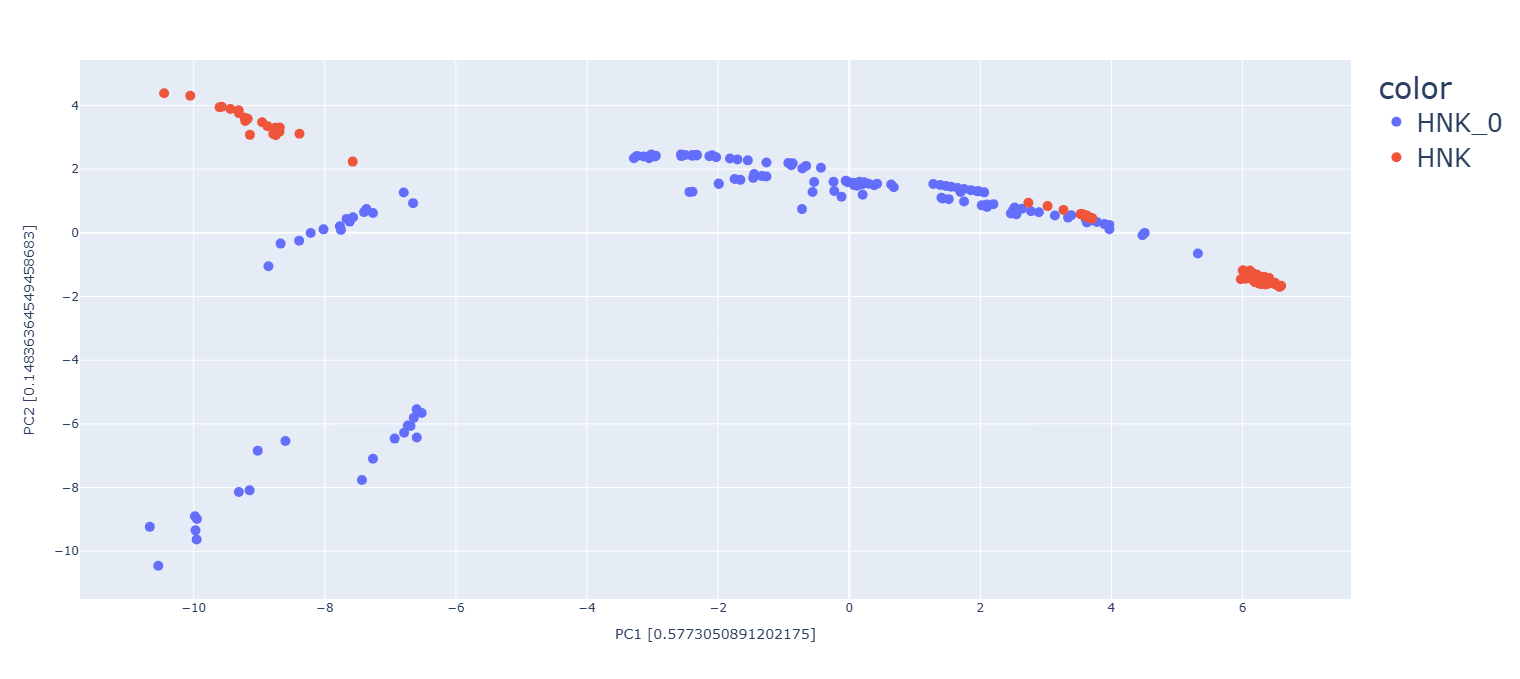

Supplement: Supplementary file 1 [file micromachines-17-00840-s001.zip › Figure S18.png]

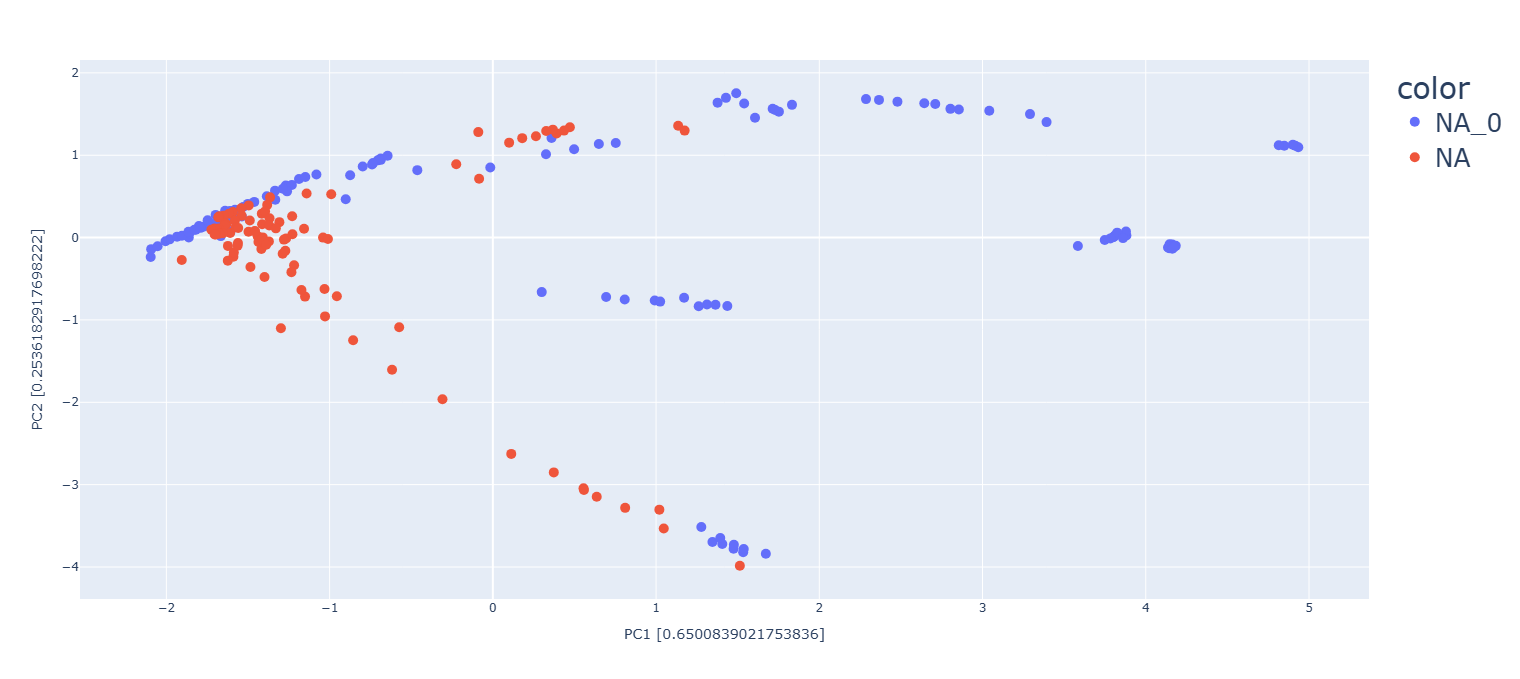

Supplement: Supplementary file 1 [file micromachines-17-00840-s001.zip › Figure S19.png]

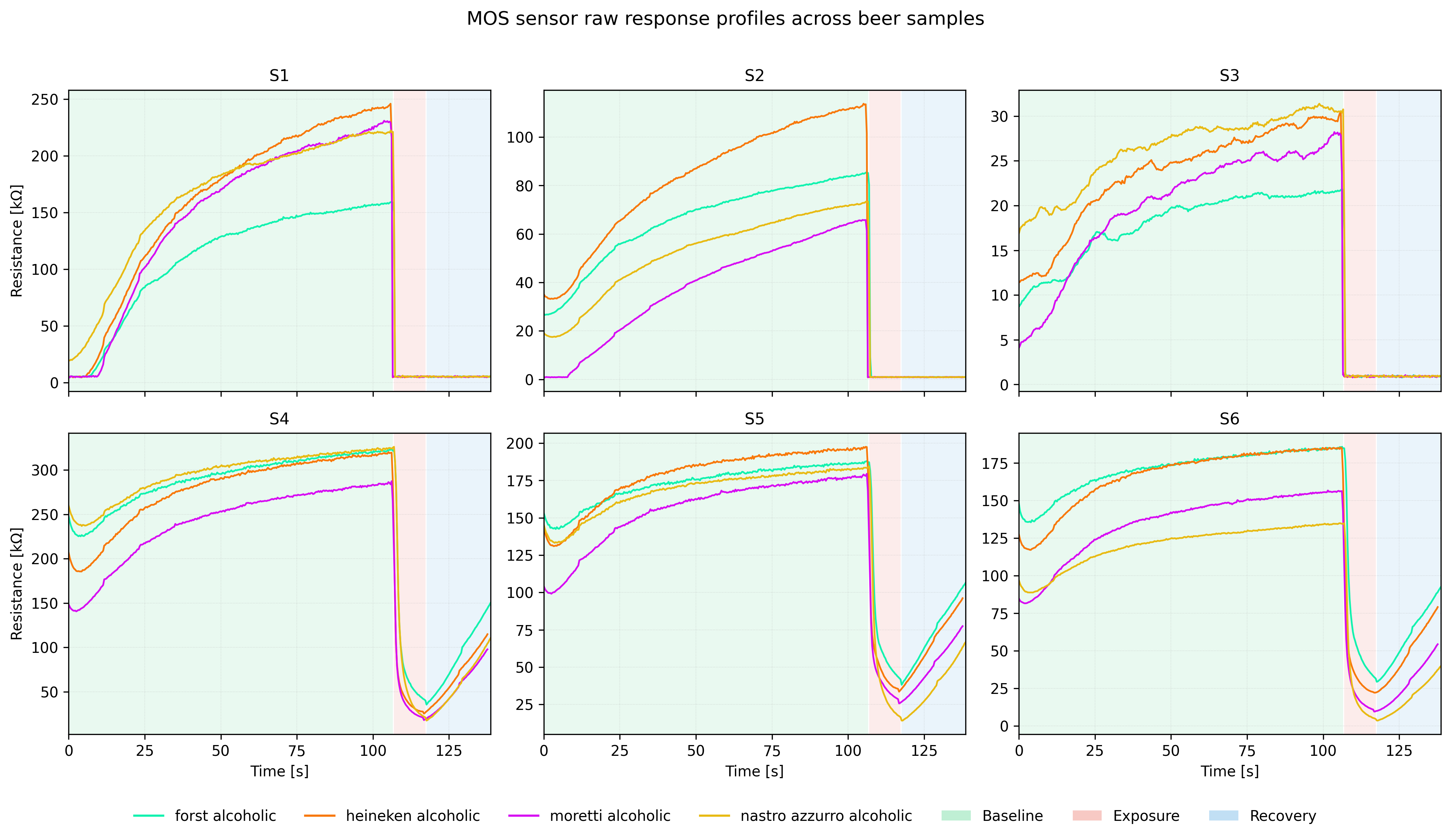

Supplement: Supplementary file 1 [file micromachines-17-00840-s001.zip › Figure S2.png]

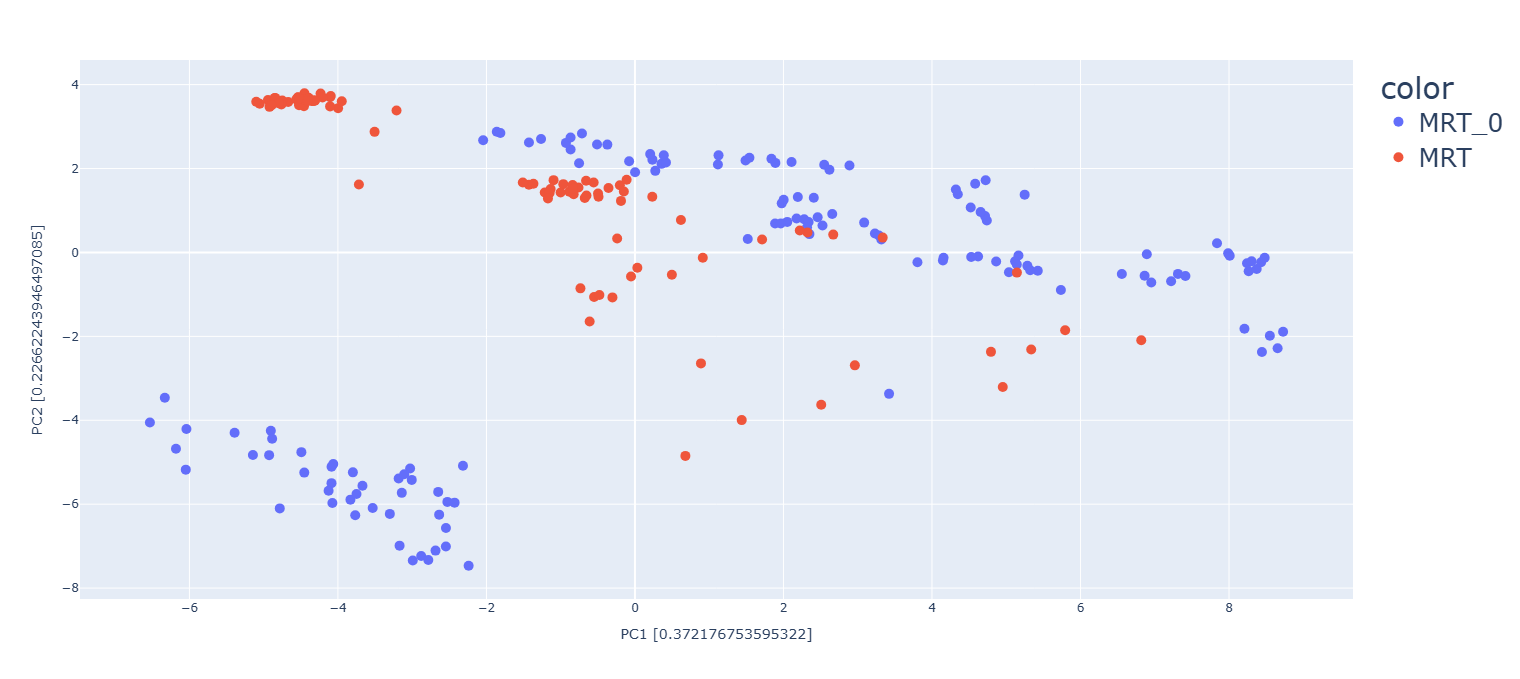

Supplement: Supplementary file 1 [file micromachines-17-00840-s001.zip › Figure S20.png]

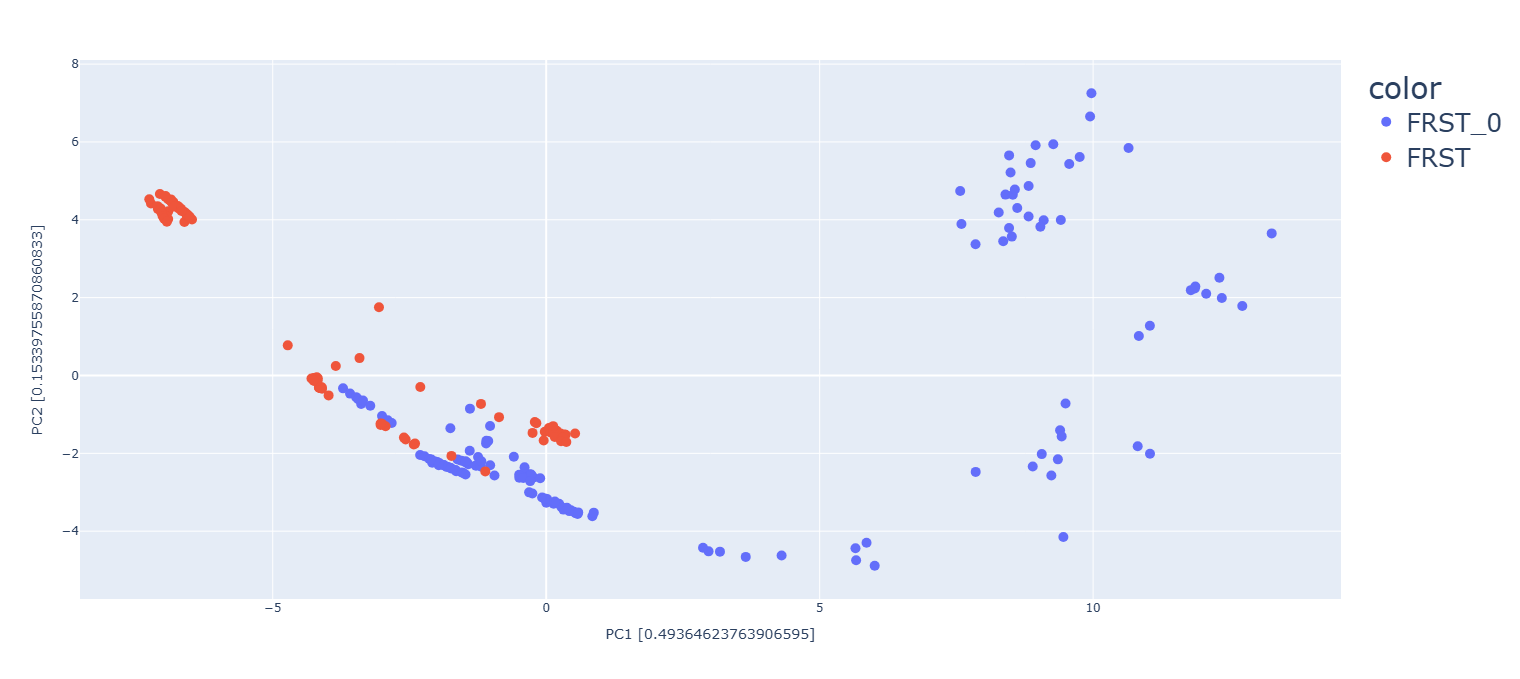

Supplement: Supplementary file 1 [file micromachines-17-00840-s001.zip › Figure S21.png]

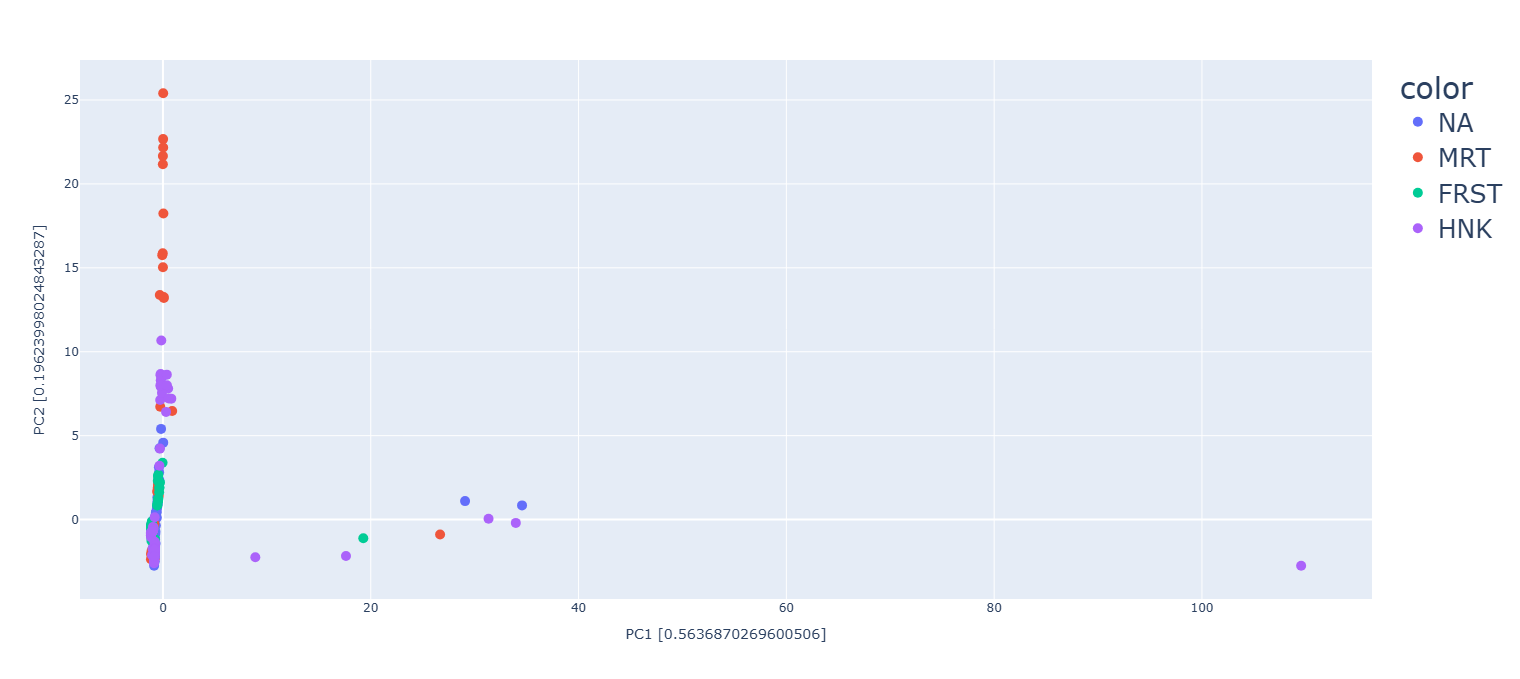

Supplement: Supplementary file 1 [file micromachines-17-00840-s001.zip › Figure S22.png]

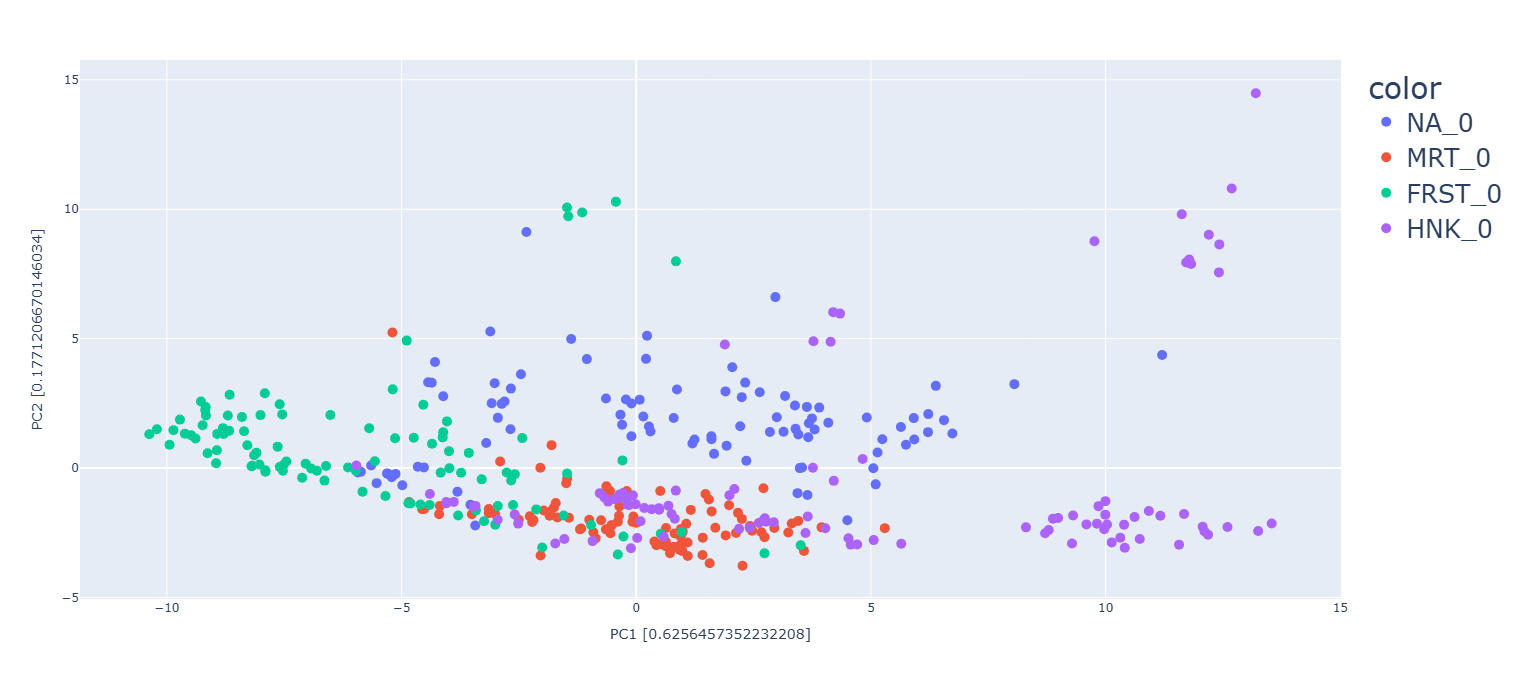

Supplement: Supplementary file 1 [file micromachines-17-00840-s001.zip › Figure S23.png]
